# Ex-vivo repair for multiple and intraparenchymal renal artery aneurysms

**DOI:** 10.1590/1677-5449.210012

**Published:** 2021-06-25

**Authors:** Prashant Jain, Anil L Naik, Azharuddin Ansari, Lileshwar Kaman, Cherring Tandup, Ujjwal Gorsi, Ajay Savlania

**Affiliations:** 1 Postgraduate Institute of Medical Education and Research – PGIMER, Chandigarh, Union Territory, India.

**Keywords:** complex renal artery aneurysm, ex-vivo repair, auto-transplantation, orthotopic, aneurisma complexo da artéria renal, reparo *ex vivo*, autotransplante ortotópico

## Abstract

A 45-year-old woman with known hypothyroidism and no other comorbidities was incidentally found to have multiple right renal artery aneurysms. The largest aneurysm measured 5 x 4.5 cm and arose from an inferior segmental branch while two smaller aneurysms arose from an upper segmental branch of the right renal artery. We performed an ex-vivo repair with reverse saphenous vein graft under cold preservation followed by orthotopic kidney auto-transplantation. Her postoperative course was unremarkable and at 1-year follow-up her right kidney is preserved. In this article, we report successful treatment of complex multiple right renal artery aneurysms and describe the surgical technique used for successful repair.

## Resumo

Uma mulher de 45 anos com hipotireoidismo conhecido e sem outras comorbidades teve achado incidental de múltiplos aneurismas da artéria renal direita. O maior aneurisma media 5 x 4,5 cm e tinha origem no ramo segmentar inferior, juntamente com dois pequenos aneurismas originários do ramo segmentar superior da artéria renal direita. Realizamos o reparo *ex vivo* com enxerto reverso de veia safena, sob preservação a frio, seguido de autotransplante renal ortotópico. O pós-operatório ocorreu sem intercorrências, e a paciente teve o rim direito preservado no seguimento de 1 ano. Neste artigo, relatamos o tratamento bem-sucedido de múltiplos aneurismas complexos da artéria renal direita e descrevemos a técnica cirúrgica utilizada para o reparo bem-sucedido.

## INTRODUCTION

Renal artery aneurysm (RAA) is a rare condition with an incidence of 0.15-1%.^1^ Most are asymptomatic and are increasingly being discovered incidentally on imaging studies performed for other reasons. Symptomatic RAAs can present with flank or abdominal pain, renovascular hypertension, ischemic nephropathy, or sometimes hematuria. Of late, endovascular techniques have offered a less invasive option, but open surgical repair with ex-vivo cold preservation techniques remains the gold standard for hilar and segmental RAAs.

## CASE REPORT

**Figure 1:**
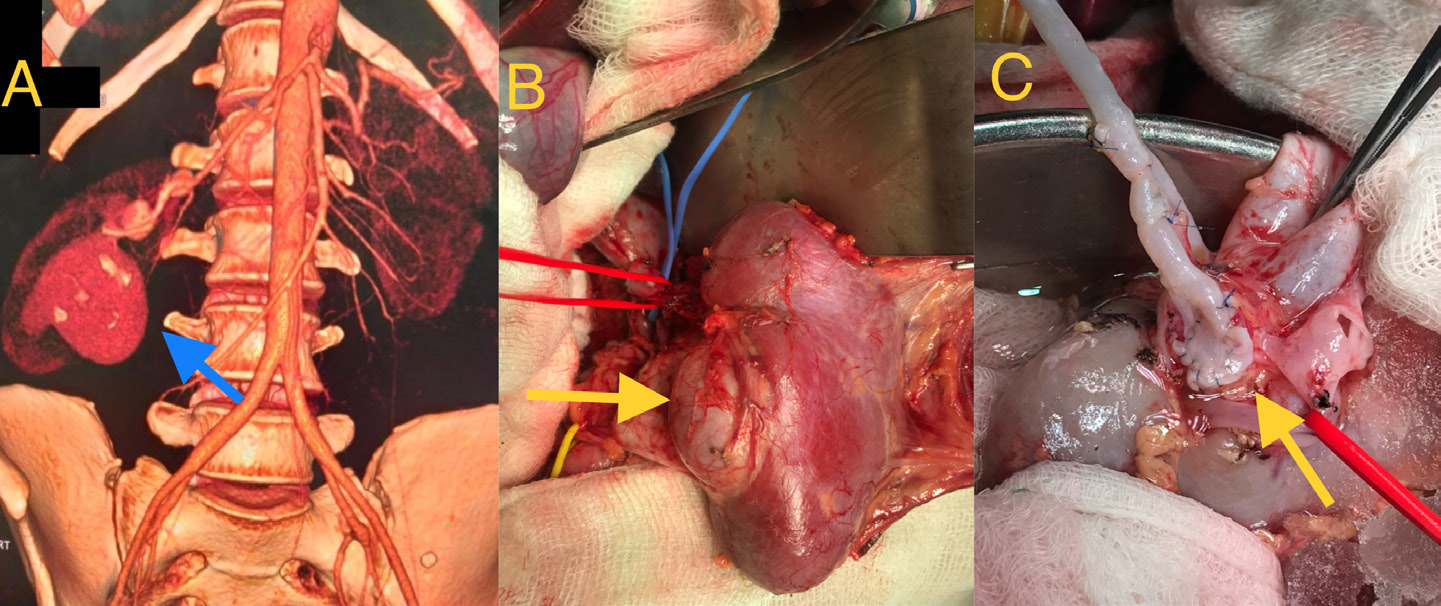
**A)** CT volume rendered image (VRT) showing multiple aneurysms in the right renal artery, the largest measuring 5 x 4.5 cm (arrow). **B)** Intraoperative picture showing the large size of the aneurysm (arrow). **C)** Intraoperative picture showing repair of aneurysm with two saphenous vein grafts.

A 45-year-old woman presented to our outpatient department with a complaint of chronic diarrhea for the past 6 months. She was normotensive and had been taking L-thyroxine for hypothyroidism for the past 13 years. During evaluation for irritable bowel syndrome, an incidental right renal artery aneurysm was found on ultrasonography of abdomen. Computed tomography (CT) angiography revealed multiple aneurysms arising from the right renal artery. The largest measured 5 x 4.5 cm in size, had a peripheral rim of calcification, and arose from the lower branch, while two smaller aneurysms arose from the upper division of the right renal artery (Figures 1A and 1B). There was no history, examination findings, or radiology findings, suggestive of intracranial, visceral, or extremity aneurysmal disease which could have indicated underlying collagen vascular disease or vasculitis.

In preoperative work-up, her blood urea and serum creatinine levels were 23.1 mg/dl and 0.7 mg/dl respectively, with normal C-reactive protein, erythrocyte sedimentation rate, and antinuclear antibody levels. The patient required intervention due to the large size of the RAA. Endovascular intervention was not feasible given the location and unfavorable anatomy. Nephrectomy was not considered as Tc 99m renal scintigraphy revealed that 54% of her renal function was derived from the right kidney.

**Figure 2:**
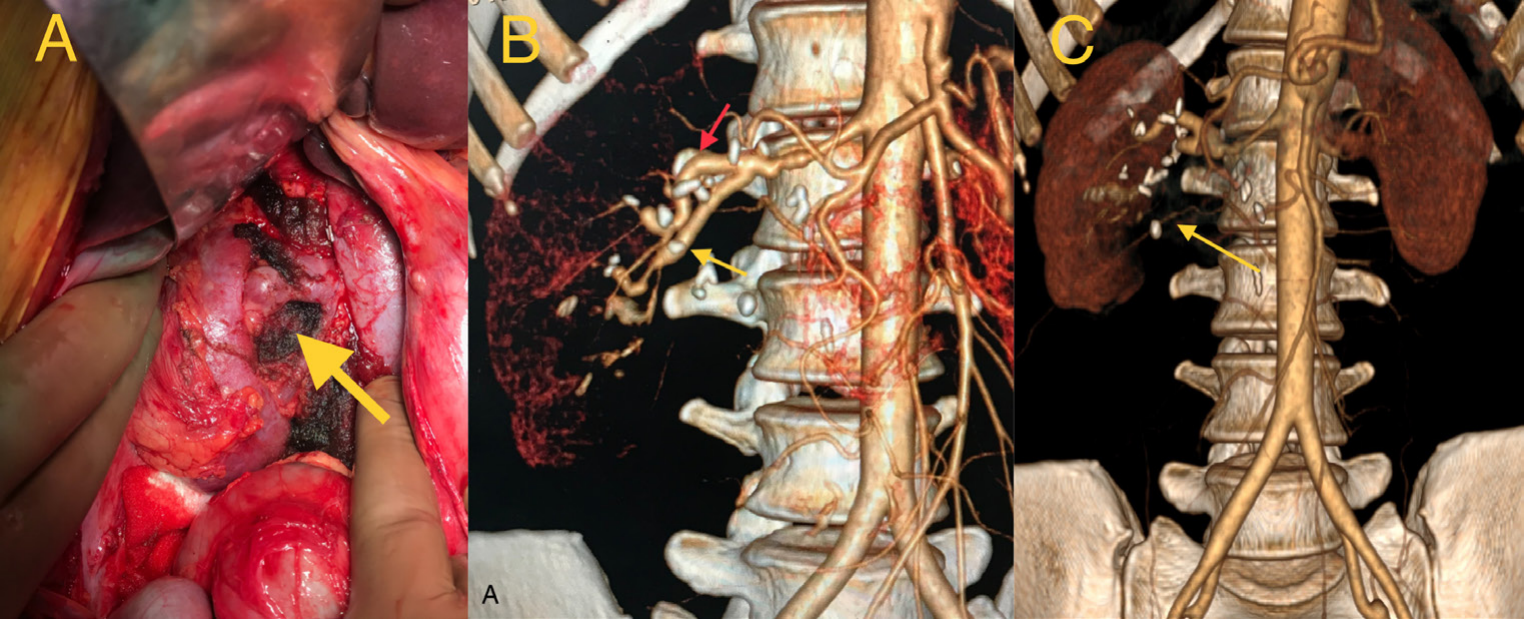
**A)** Picture showing well-perfused, pink colored, right kidney in its bed after orthotopic auto transplantation. **B)** Postoperative CT VRT images at 1-year follow-up, showing intact repair of aneurysm. **C)** Delayed CT image showing well-perfused renal parenchyma.

She was scheduled for surgical repair of right RAA. Under general anesthesia, a right subcostal incision was used for unilateral renal artery exposure. Extended kocherization was performed, the right kidney was mobilized optimally, so that it could be kept ex vivo with ureter intact; systemic heparin 1mg/kg was given, and the renal vein was divided with a cuff of inferior vena cava (IVC) along with the proximal renal artery. The kidney was brought out of the abdomen and placed in ice slush. Cold renal preservation was achieved by intermittent flushing with Custodial histidine-tryptophan-ketoglutarate solution. An autogenous great saphenous graft was chosen as conduit. After cooling was complete, the aneurysm (approximately 5 x 4.5 cm) involving the inferior segmental artery was opened and outflow vessels were identified. The renal parenchyma was not opened as the lumens of all outflow vessels were clearly visualized and were accessible to fashion the distal anastomosis. The harvested venous graft was configured accordingly and distal anastomosis was done in the sac of large aneurysm over the outflow ends and another vein graft was used to anastomose it with the upper division distal to the small aneurysms (Figure 1C). Once reconstruction was complete, the kidney was placed back in the renal fossa, the vein graft was anastomosed with the end of the right renal artery, and the renal vein was anastomosed with the IVC. The kidney became pink once flow was reestablished (Figure 2A) and flow was further confirmed with handheld doppler.

The patient recovered unremarkably. In the postoperative period, she was normotensive and serum urea and creatinine levels were 12 mg/dl and 0.6 mg/dl respectively. Histopathologic examination of the aneurysm sac wall showed cystic medial degeneration and there were no features suggestive of any connective tissue disorders or vasculitis. Her postoperative CT angiogram at 1 year follow up showed an intact repair and well-perfused right kidney (Figure 2B and 2C).

## DISCUSSION

Complex hilar renal artery aneurysm (RAA) is a rare but surgically challenging condition. The incidence of simultaneous non-renal artery aneurysms (i.e., aortic, iliac, and visceral) is 7%-30%.^2^ The most common sites include the carotid and intracranial arteries.^3^ In a multi-institutional review of 865 RAAs, extra-renal aneurysms involved the abdominal aorta (37 patients), splenic artery (23 patients), celiac artery (5 patients), and hepatic artery (4 patients).^4^ RAAs may occur due to connective tissue disorders in younger patients or atherosclerosis in the elderly.^1^

Most RAAs are asymptomatic, but recent reports have emphasized their roles in manifestation of secondary hypertension. According to consensus, the indications for repair of RAAs are diameter >2cm, renovascular hypertension, intrarenal thromboembolism, and lesions in women of childbearing age.^2^

Many options for repair of RAAs are available depending on the location and complexity of the aneurysm. Open surgery with either an ex vivo or in situ approach is preferred for complex hilar or intraparenchymal RAAs. Further, auto-transplantation can be done within the renal fossa or into the iliac fossa. The latter approach requires the additional complexity of ureteroneocystostomy and future atherosclerosis of the iliac artery can affect perfusion to the kidney. Also, unlike transplant patients, these patients will not require an access for biopsy or ease of removal in case of rejection or pain.^5^^,^^6^ Therefore, the index case with 3 aneurysms (the largest from the lower division with intrarenal extension) was successfully managed with ex vivo repair and orthotopic auto-transplantation in the right renal fossa. Endovascular techniques like stent graft placement or coil embolization are reserved for more proximal aneurysms with a narrow neck.^7^^,^^8^

A warm ischemia time of <30 minutes is usually well tolerated by the kidneys and for longer duration surgeries, perfusion of a cold preservative solution is generally required, as described in the present case.

The present case report is in accordance with the Indian Council of Medical Research guidelines and the Helsinki Declaration. Written informed consent was obtained from the patient and approval was granted by an independent departmental scientific screening committee at the Postgraduate Institute of Medical Education and Research, Chandigarh, India.

## CONCLUSION

Ex-vivo surgical repair is a successful and durable treatment for complex distal RAAs. It has low morbidity, mortality and provides good renal function preservation.
